# Supplementary material for: Changes in Air Quality during the Period of COVID-19 in China
Source: Int J Environ Res Public Health. 2022 Dec 2;19(23):16119. doi: 10.3390/ijerph192316119 (PMC9737528; doi:10.3390/ijerph192316119)
Supplement: Supplementary file 1 [file ijerph-19-16119-s001.zip › ijerph-1926617-supplementary.pdf]

Supplementary Materials for

Changes in air quality during the period of COVID-19 in China

This file includes:

Figure S1. The trends in the mean values of the last IMFs in each month
Figure S2. The trends in percentage of cities with improved air quality
Figure S3. The trends in monthly mean values of the last IMFs in 12 cities
Figure S4. The changes of evolution trends of AQI of the last IMFs in 12 cities
Figure S5. Evolution of AQI trends in 24 cities
Figure S6. Evolution of AQI trends during different periods
Figure S7. Trends in the last IMFs
Figure S8. Temporal trends in AQI in 12 cities experiencing severe COVID-19 epidemic conditions
Figure S9. Recurrence plots of the evolution of trends in AQI from Jan 1, 2018, to Dec 27, 2020, in cities that experienced severe epidemic conditions
Figure S10. Temporal trends in AQI in 12 cities with few COVID-19 cases
Figure S11. The recurrence plot of the evolution of trends in AQI from Jan 1, 2018, to Dec 27, 2020, in the cities where the epidemic was not serious
Figure S12. Temporal trends in AQI in cities with secondary outbreaks
Figure S13. Recurrence plot of the evolution of trends in AQI in cities with secondary outbreaks
Figure S14. Time trends of the Monthly city-wide mean value for different primary pollutants
Figure S15 Histogram of RQA indicators of 24 cities
Figure S16 The trends of AQI in monthly mean values of the last IMFs in different cities by adopting CEEMDAN method
Figure S17 The trends of six pollutants in monthly mean values of the last IMFs in different cities by adopting CEEMDAN method
Table S1 Monthly city-wide mean value for different primary pollutants for 2018
Table S2 Monthly city-wide mean value for different primary pollutants for 2019
Table S3 Monthly city-wide mean value for different primary pollutants for 2020
The explanation of the definition of daily AQI and its formula
The explanation of EEMD and CEEMDAN

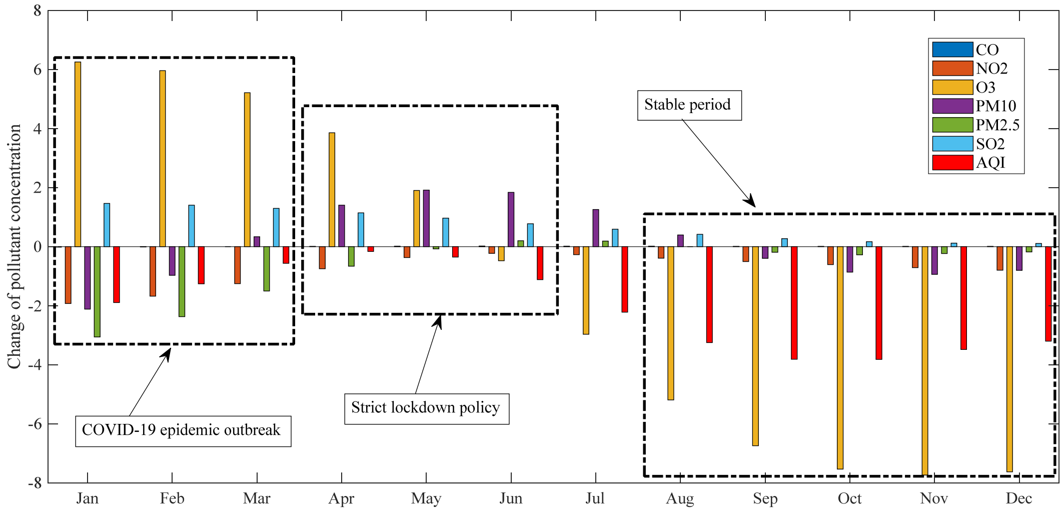

Figure S1. The trends in the mean values of the last IMFs in each month

Notes: This fig reports different trends in the mean values of the last IMFs of different air pollutants at different levels of outbreaks and lockdown measures.

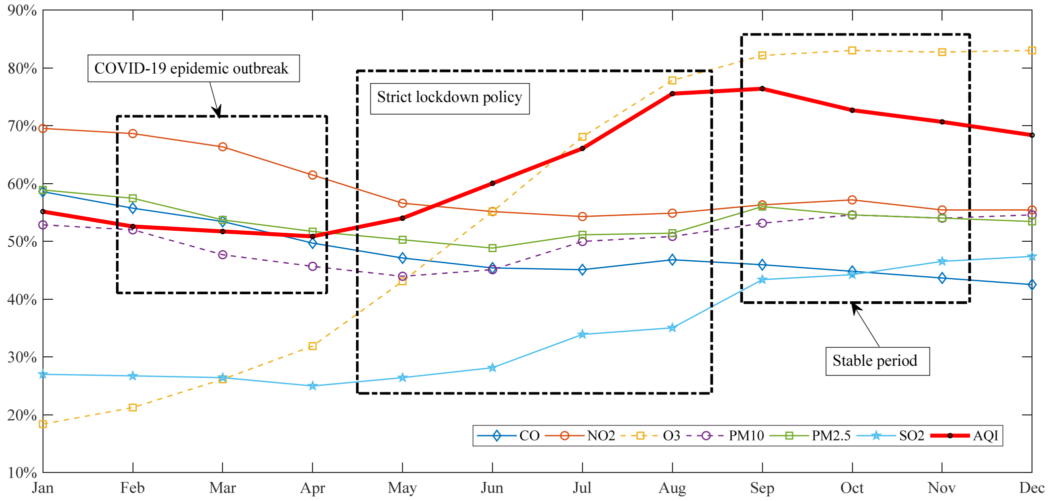

Figure S2. The trends in percentage of cities with improved air quality

Notes: Figure S2 counted the percentage of cities with improved air quality each month.

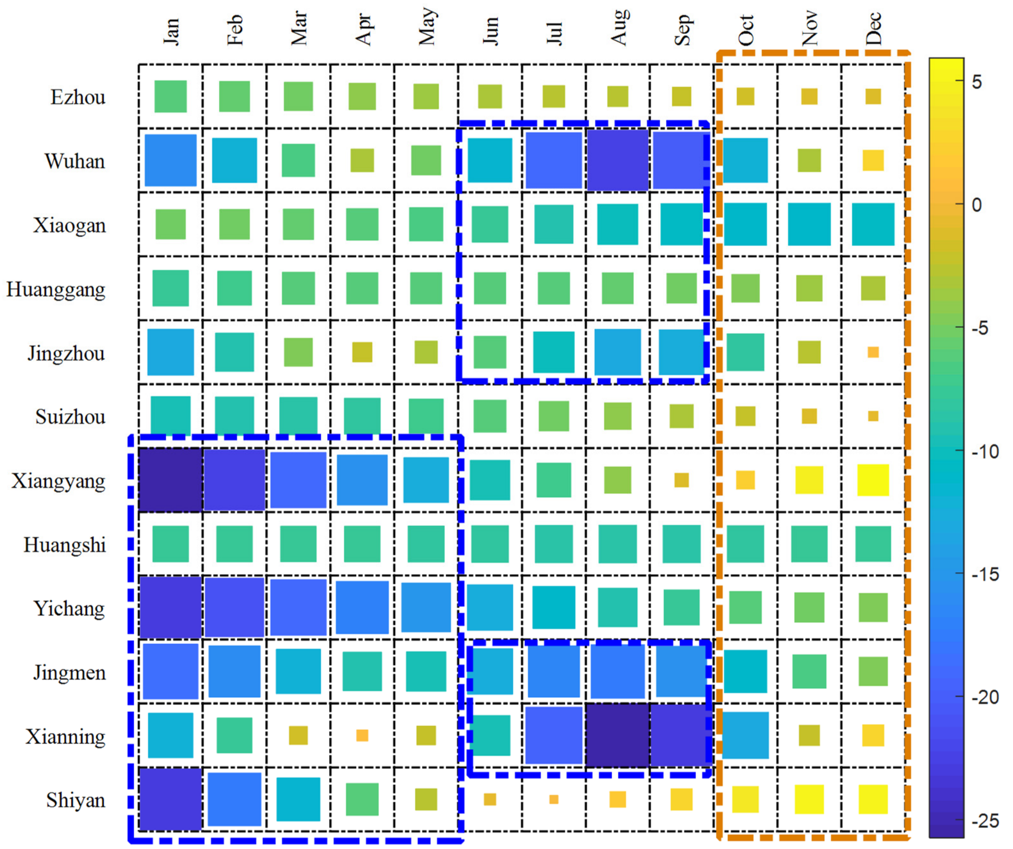

Figure S3. The trends in monthly mean values of the last IMFs in 12 cities

Notes: In Figure S3, the color of each square corresponds to its size, and the closer the color is to blue, the smaller the value. The larger the square, the greater the absolute value.

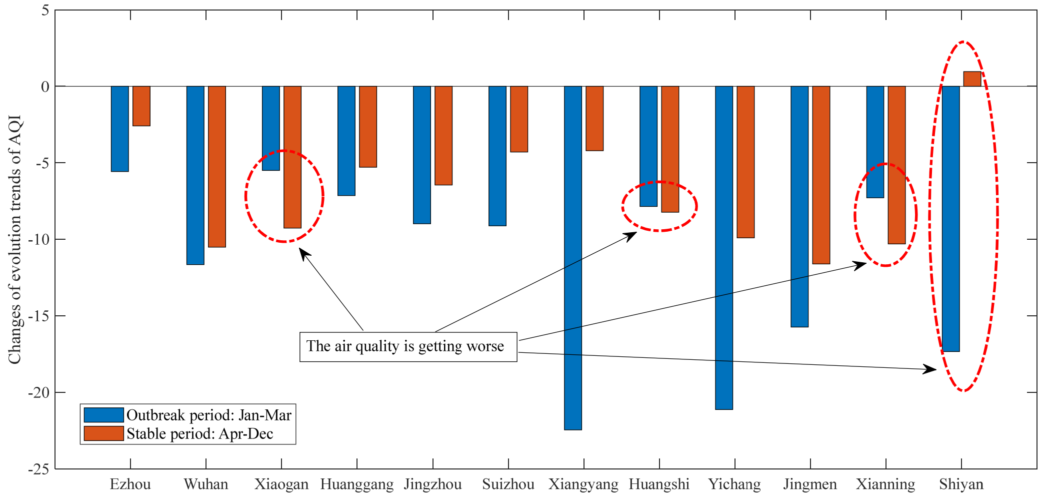

Figure S4. The changes of evolution trends of AQI of the last IMFs in 12 cities

Notes: In Figure S4, the blue bars represent the change in AQI during the outbreak period, from January to March. The red bars represent periods of stability that cities have experienced since the outbreak, from April to December.

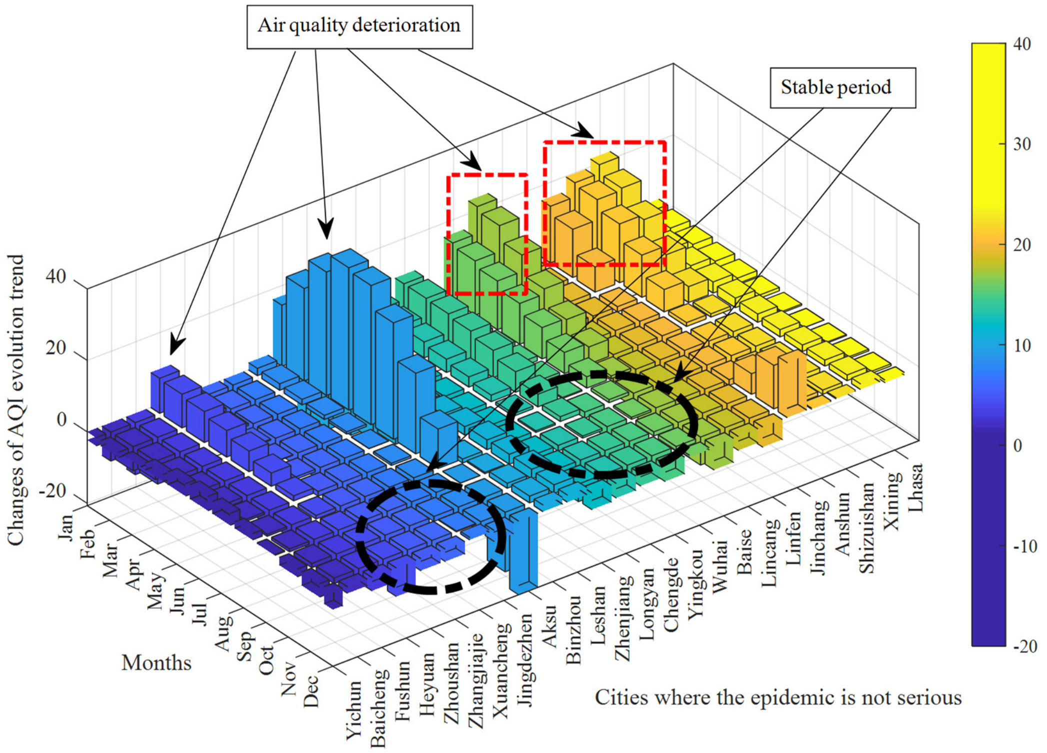

Figure S5. Evolution of AQI trends in 24 cities

Notes: In Figure S5, a value greater than 0 indicates poor air quality, while a value less than 0 indicates good air quality, corresponding to blue and yellow respectively.

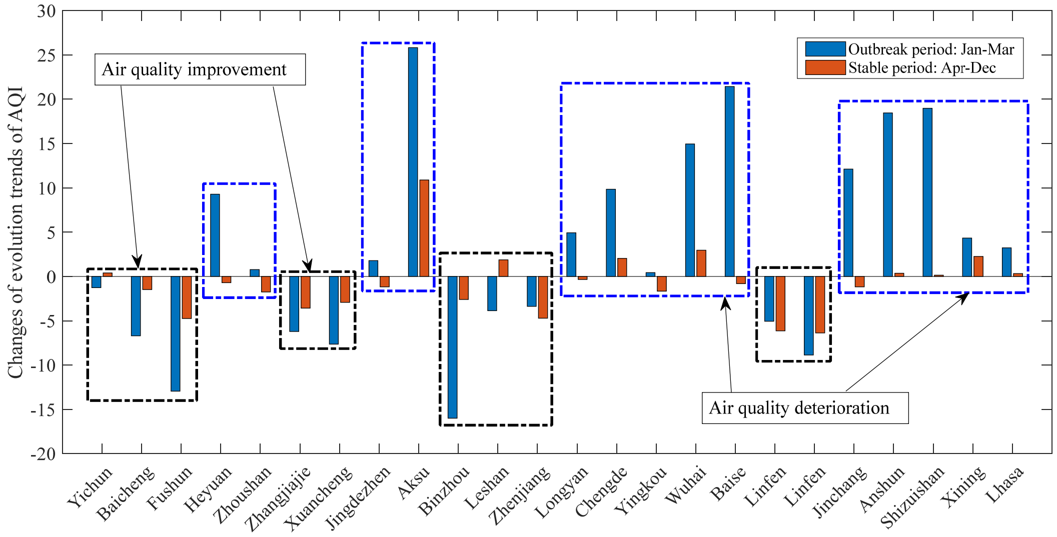

Figure S6. Evolution of AQI trends during different periods

Notes: In Figure S6, the blue bars represent the change in AQI during the outbreak period, from January to March. The red bars represent periods of stability that cities have experienced since the outbreak, from April to December.

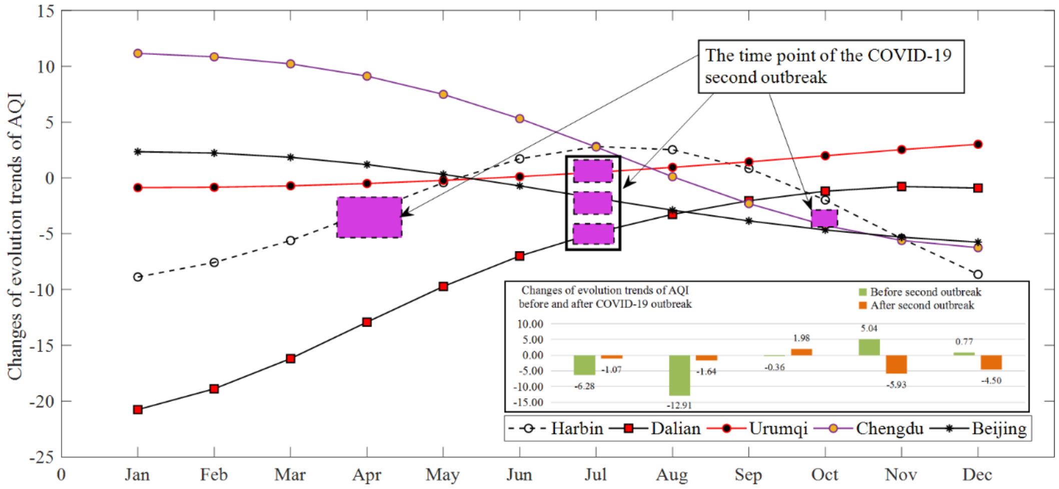

Figure S7. Trends in the last IMFs

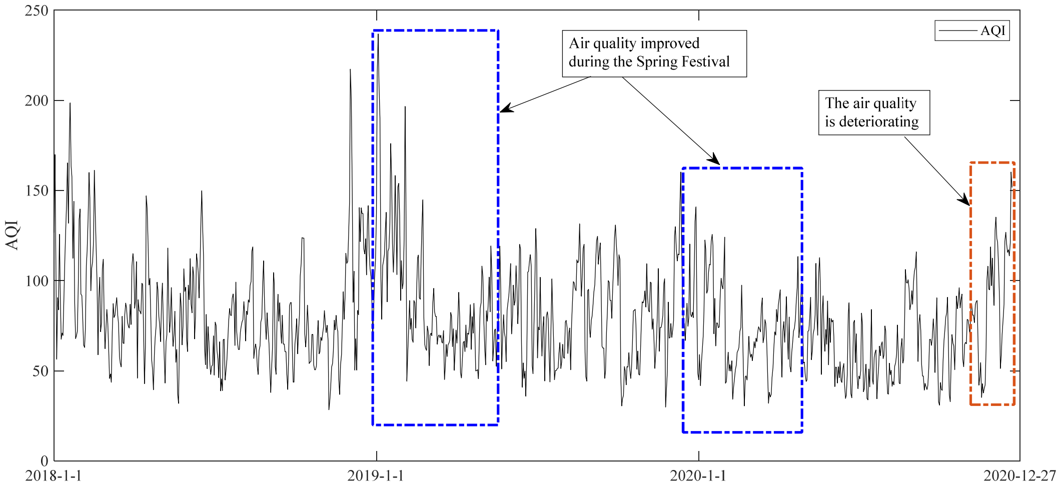

Figure S8. Temporal trends in AQI in 12 cities experiencing severe COVID-19 epidemic conditions

As shown in *Supplementary Materials*, Figure S8, air quality deteriorated first and then improved. After adopting the phase space reconstruction to analyze the last IMF, the recurrence plot could be constructed (Shown in *Supplementary Materials*, Figure S9), depicting the changes in characteristics of long-term trends in AQI.

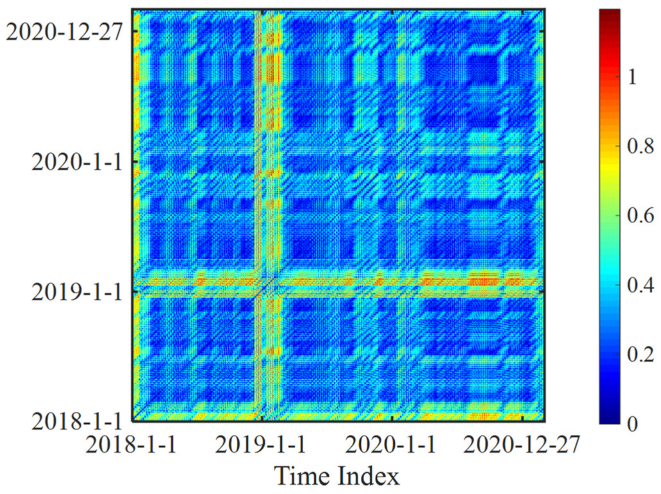

Figure S9. Recurrence plots of the evolution of trends in AQI from Jan 1, 2018, to Dec 27, 2020, in cities that experienced severe epidemic conditions

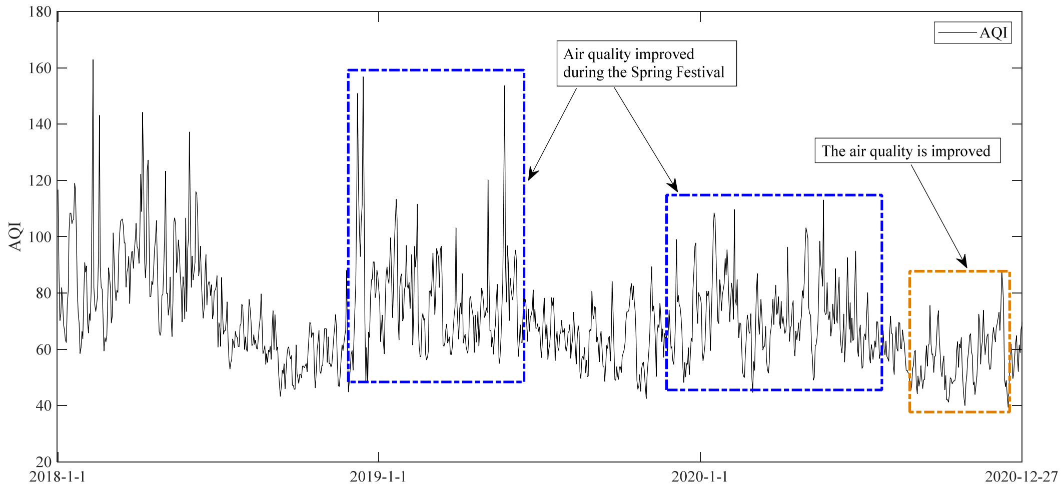

Figure S10. Temporal trends in AQI in 12 cities with few COVID-19 cases

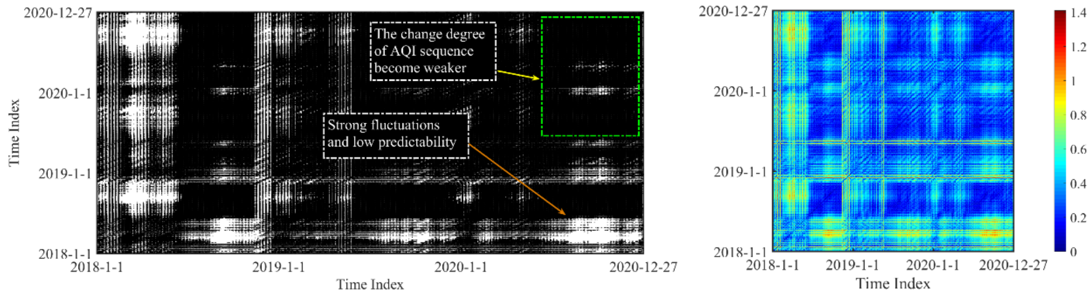

(a) Black-and-white recursive graph (b) Color recursive graph

Figure S11. The recurrence plot of the evolution of trends in AQI from Jan 1, 2018, to Dec 27, 2020, in the cities where the epidemic was not serious

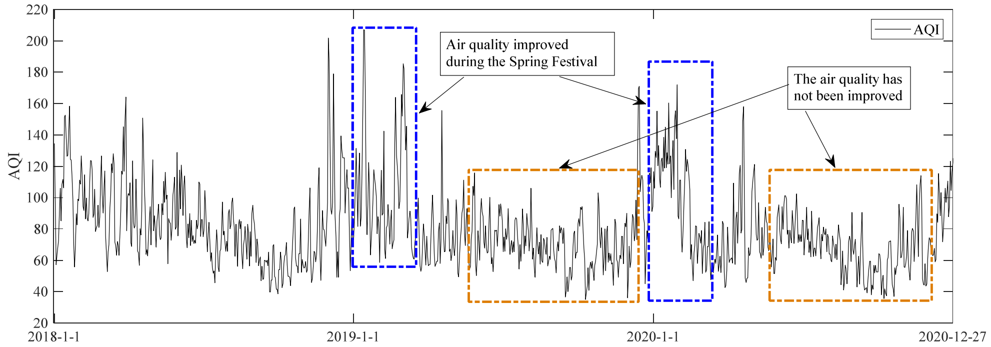

Figure S12. Temporal trends in AQI in cities with secondary outbreaks

In the cities that experienced secondary outbreaks, the air quality showed a pattern of initial improvement and subsequent deterioration (*Supplementary Materials*, Figure S12). It could be deduced that in the first stage of the epidemic, the strict travel restriction policy inhibited the residents' movement and reduced the residents' consumption levels. Hence, the emissions of various air pollutants showed downward trends.

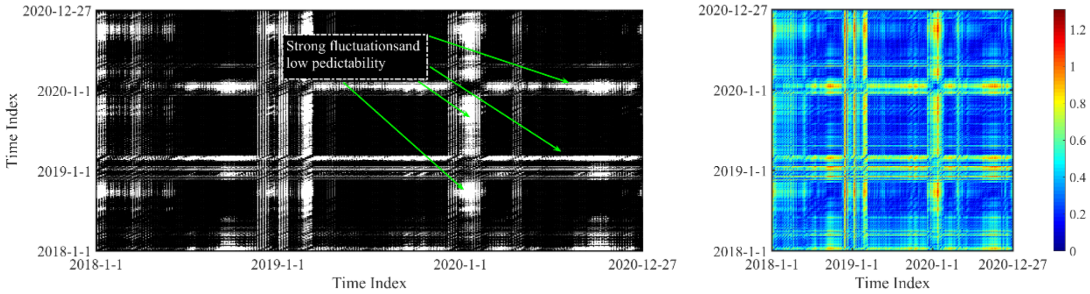

(a) Black-and-white recursive graph (b) Color recursive graph

Figure S13. Recurrence plot of the evolution of trends in AQI in cities with secondary outbreaks

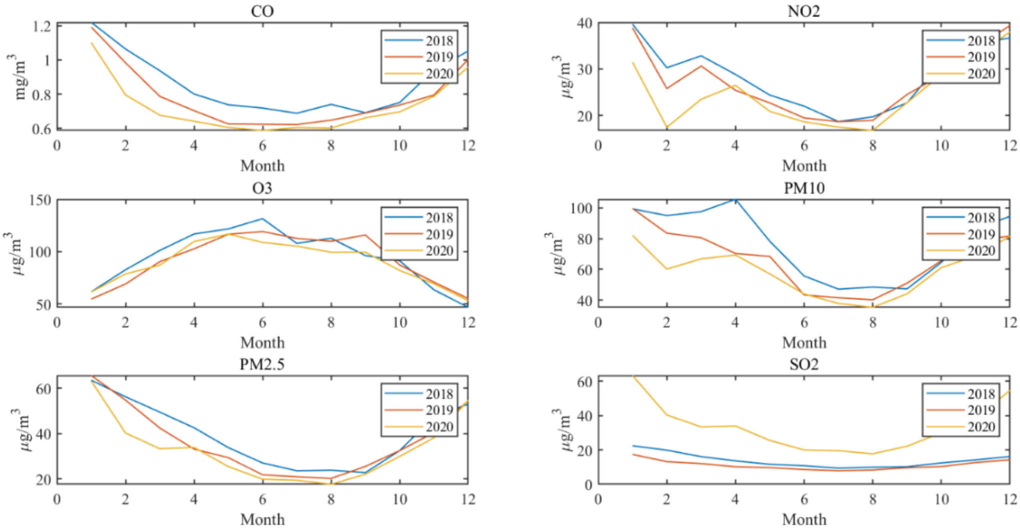

Figure S14. Time trends of the Monthly city-wide mean value for different primary pollutants

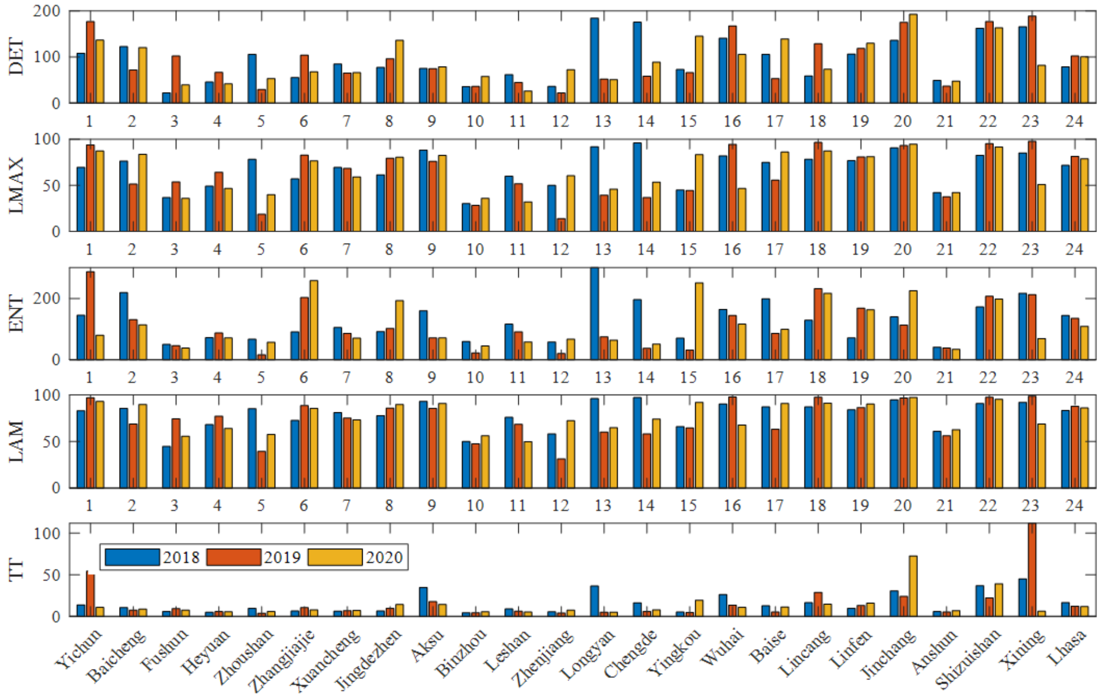

Figure S15 Histogram of RQA indicators of 24 cities

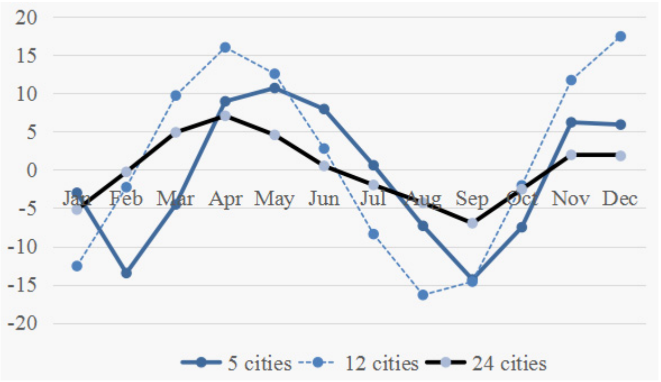

Figure S16 The trends of AQI in monthly mean values of the last IMFs in different cities by adopting CEEMDAN method

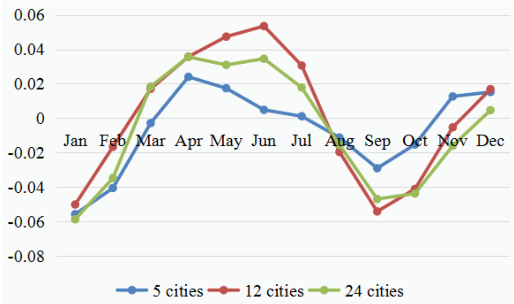

(a) CO

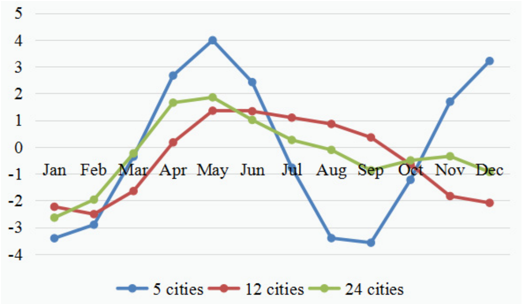

(b) NO2

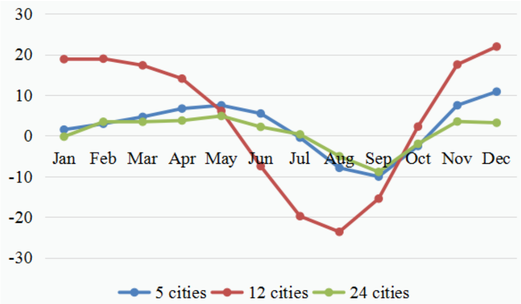

(c) O3

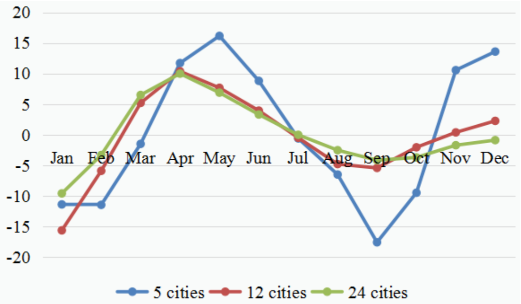

(d) PM10

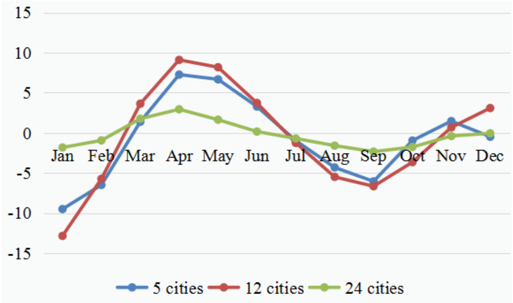

(e) PM2.5

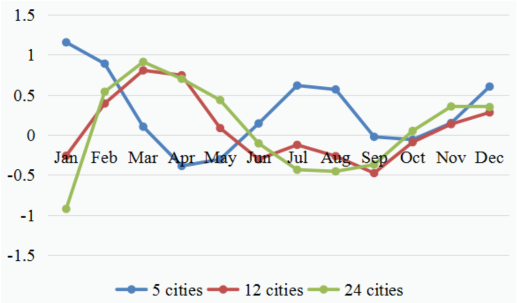

(f) SO2

Figure S17 The trends of six pollutants in monthly mean values of the last IMFs in different cities by adopting CEEMDAN method

Table S1 Monthly city-wide mean value for different primary pollutants for 2018

Month	CO (mg/m^3)	NO_2 ($\mu g/m^3$)	O_3 ($\mu g/m^3$)	PM_{10} ($\mu g/m^3$)	$PM_{2.5}$ ($\mu g/m^3$)	SO_2 ($\mu g/m^3$)
1	1.2198	39.6106	61.7278	99.3354	63.5933	22.4028
2	1.0643	30.2436	82.7567	94.9469	56.2764	19.8391
3	0.9375	32.8058	101.2128	97.5161	49.5255	16.0792
4	0.8012	28.8112	116.8806	105.5722	42.6285	13.6261
5	0.7379	24.3632	121.7703	78.2400	33.8845	11.6260
6	0.7191	21.9832	131.4798	55.6359	27.1166	10.7813
7	0.6886	18.6657	107.9148	47.0563	23.6599	9.3598
8	0.7406	19.6922	112.8896	48.4041	23.9332	9.9229
9	0.6916	22.6980	96.3192	47.2625	22.8438	10.2004
10	0.7513	31.5621	91.3409	64.5056	32.5513	12.4210
11	0.9405	35.5959	63.6818	86.0098	47.4035	14.1726
12	1.0523	36.6235	46.9113	94.5350	53.0552	16.1191

Table S2 Monthly city-wide mean value for different primary pollutants for 2019

Month	CO (mg/m^3)	NO_2 ($\mu g/m^3$)	O_3 ($\mu g/m^3$)	PM_{10} ($\mu g/m^3$)	$PM_{2.5}$ ($\mu g/m^3$)	SO_2 ($\mu g/m^3$)
1	1.1925	38.8315	54.7390	99.6047	65.6468	17.3702
2	0.9837	25.7725	69.1726	83.5105	54.8176	13.1658
3	0.7875	30.6144	90.5057	80.5658	42.5119	12.0328
4	0.7036	25.3971	102.7559	70.2874	33.2397	10.2034
5	0.6259	22.6801	116.6257	68.3803	29.4489	9.6551
6	0.6241	19.4559	119.2433	43.2856	21.9006	8.5884
7	0.6226	18.6550	112.6791	41.4397	21.0164	7.8487
8	0.6483	18.9220	109.9233	40.1567	20.3108	8.3028
9	0.6900	24.4950	115.9742	50.9717	25.5830	9.6327
10	0.7362	28.7057	87.3637	65.5792	32.5437	10.2998
11	0.7953	34.3833	71.0088	78.5536	40.5004	12.6607
12	1.0029	39.3632	55.3291	81.6577	54.6078	14.2308

Table S3 Monthly city-wide mean value for different primary pollutants for 2020

Month	CO (mg/m^3)	NO_2 ($\mu g/m^3$)	O_3 ($\mu g/m^3$)	PM_{10} ($\mu g/m^3$)	$PM_{2.5}$ ($\mu g/m^3$)	SO_2 ($\mu g/m^3$)
1	1.1006	31.4249	61.6027	82.0189	63.2799	13.6146
2	0.7962	17.4440	78.7700	60.1109	40.3147	10.2671
3	0.6774	23.4652	87.1284	66.7904	33.3864	9.8687
4	0.6417	26.4554	109.7008	69.2933	33.9515	10.2320
5	0.6059	20.8597	116.8038	56.9889	25.5278	8.9378
6	0.5881	18.6128	108.9033	43.9142	19.9626	8.1626
7	0.6056	17.4845	105.2846	37.7640	19.5135	7.6451
8	0.6025	16.7592	99.4865	35.2063	17.7312	7.7978
9	0.6621	22.7543	99.5111	44.0435	22.1381	8.6136
10	0.6968	28.7740	82.1234	61.0899	30.0165	10.1025
11	0.7884	32.9779	69.4292	68.6248	38.2082	11.5769
12	0.9586	37.9347	53.3517	81.5431	54.6730	13.7312

The explanation of the definition of daily AQI and its formula

The air quality index (AQI) is calculated by

$$AQI = \max\{IAQI_1, IAQI_2, IAQI_3, \dots, IAQI_n,\} \quad (1)$$

Where $IAQI_p$ is the air quality sub index, such as $PM_{2.5}$ and O_3, and $IAQI_p$ is obtained by

$$IAQI_p = \frac{IAQI_{H_i} - IAQI_{L_o}}{BP_{H_i} - BP_{L_o}} \left(C_p - BP_{L_o}\right) + IAQI_{L_o} \quad (2)$$

Where $IAQI_p$ indicates the air quality sub index of pollutant p. C_p is the

concentration value of pollutant p . BP_{H_i} is the high value of the pollutant concentration limit similar to C_p in Table S4. BP_{L_o} is the low value of the pollutant concentration limit similar to C_p in Table S4. $IAQI_{H_i}$ is the air quality sub index corresponding to BP_{H_i} in Table S4. $IAQI_{L_o}$ is the air quality sub index corresponding to BP_{L_o} in Table S4. Table S4 is shown as follows.

Table S4 Introduction for pollutant concentration limit

IAQI	Pollutant concentration limit					
	SO_2 24-hour average ($\mu g/m^3$)	NO_2 24-hour average ($\mu g/m^3$)	PM_{10} 24-hour average ($\mu g/m^3$)	CO 24-hour average ($\mu g/m^3$)	O_3 8-hour average ($\mu g/m^3$)	$PM_{2.5}$ 24-hour average ($\mu g/m^3$)
0	0	0	0	0	0	0
50	50	40	50	2	100	35
100	150	80	150	4	160	75
150	475	180	250	14	215	115
200	800	280	350	24	265	150
300	1600	565	420	36	800	250
400	2100	750	500	48	(1)	350
500	2620	940	600	60	(1)	500

Note: (1) When the average concentration of O_3 8-hour average ($\mu g/m^3$) is greater than 800 ($\mu g/m^3$), its air quality sub index is no longer calculated and reported according to O_3 1-hour average ($\mu g/m^3$), namely 1000 and 1200.

The explanation of EEMD and CEEMDAN

EEMD could make up the shortcoming of EMD. In the process of decomposing the IMF with EMD, many iterations are required, and the conditions for stopping the iteration lack a standard, so the IMFs obtained by different conditions for stopping the iteration are also different (Qiu et al., 2022). This shortcomings has been solved by EEMD proposed by Wu and Huang (2009) and used in this paper. In addition, EEMD

can effectively solve the mode aliasing problem caused by EMD, and it can accurately and efficiently divide different frequency components. Specifically, when EEMD is adopted, white noise is added to the signal to be analyzed by taking advantage of the characteristic of uniform spectrum distribution of white noise, so that signals with different time scales can be automatically separated to the corresponding reference scale, which is the EEMD method. This method mainly adds white noise to the signal to supplement some missing scales, and has a good performance in signal decomposition. The EEMD decomposition principle is: when the additional white noise is evenly distributed in the whole time-frequency space, the time-frequency space is composed of different scale components divided by the filter bank.

CEEMDAN can further solve the problem of mode aliasing. Firstly, the IMF component with auxiliary noise after EMD decomposition is added in this method, instead of adding Gaussian white noise signal directly to the original signal. Secondly, EEMD decomposition take the overall average of the modal components obtained after the empirical mode decomposition, while CEEMDAN decomposition takes the overall average calculation after the first order IMF component, and obtains the final first order IMF component. Then, the above operations are repeated for the residual parts. In this way, the transfer of white noise from high frequency to low frequency is effectively solved.
